# Circadian Variation of the Human Metabolome Captured by Real-Time Breath Analysis

**DOI:** 10.1371/journal.pone.0114422

**Published:** 2014-12-29

**Authors:** Pablo Martinez-Lozano Sinues, Leila Tarokh, Xue Li, Malcolm Kohler, Steven A. Brown, Renato Zenobi, Robert Dallmann

**Affiliations:** 1 Department of Chemistry and Applied Biosciences, ETH Zurich, Zurich, Switzerland; 2 Institute of Pharmacology and Toxicology, University of Zurich, Zurich, Switzerland; 3 University Hospital of Child and Adolescent Psychiatry and Psychotherapy, University of Bern, Bern, Switzerland; 4 Pulmonary Division, University Hospital Zurich, Zurich, Switzerland; 5 Zurich Center for Integrative Human Physiology, University of Zurich, Zurich, Switzerland; McGill University, Canada

## Abstract

Circadian clocks play a significant role in the correct timing of physiological metabolism, and clock disruption might lead to pathological changes of metabolism. One interesting method to assess the current state of metabolism is metabolomics. Metabolomics tries to capture the entirety of small molecules, i.e. the building blocks of metabolism, in a given matrix, such as blood, saliva or urine. Using mass spectrometric approaches we and others have shown that a significant portion of the human metabolome in saliva and blood exhibits circadian modulation; independent of food intake or sleep/wake rhythms. Recent advances in mass spectrometry techniques have introduced completely non-invasive breathprinting; a method to instantaneously assess small metabolites in human breath. In this proof-of-principle study, we extend these findings about the impact of circadian clocks on metabolomics to exhaled breath. As previously established, our method allows for real-time analysis of a rich matrix during frequent non-invasive sampling. We sampled the breath of three healthy, non-smoking human volunteers in hourly intervals for 24 hours during total sleep deprivation, and found 111 features in the breath of all individuals, 36–49% of which showed significant circadian variation in at least one individual. Our data suggest that real-time mass spectrometric "breathprinting" has high potential to become a useful tool to understand circadian metabolism, and develop new biomarkers to easily and in real-time assess circadian clock phase and function in experimental and clinical settings.

## Introduction

Biological clocks help most organisms to anticipate daily changes in their environment that are ultimately due to the rotation of the earth. In mammals, a master clock in the brain, i.e., the suprachiasmatic nuclei (SCN), synchronizes the body to the environment and at the same time the master clock in the SCN is part of a complex web of interaction with other so-called peripheral clocks in other organs and tissues. In fact, the clock mechanism is a cell-autonomous transcriptional-(post-)translational feedback loop that is present in most if not all cells of the body (reviewed in [1]).

Most of human physiology is modulated by the circadian clock depending on time of day (reviewed in [2]). This has far reaching consequences for human health and disease. Not only can pathologies disrupt the finely tuned internal synchrony, but disruption of the circadian clock can also have negative health consequences, as reported in animal models as well as epidemiological studies. The pathologies associated with clock malfunction range from psychiatric illness to metabolic syndrome and cancer. Schizophrenics, for example, often have disrupted sleep/wake cycles [3], [4] while sleep disruption and circadian misalignment in shift-workers have been shown to contribute to obesity and diabetes as well as an increased risk for cancer [5]–[9]. These epidemiological observations are backed up by mouse models of – on the one hand – various diseases that show blunted rhythms and – on the other hand – circadian disruption like jet-lag that lead to multiple pathologies [6], [10]–[13].

After the circuitry and molecular mechanisms underlying the circadian clockwork were largely elucidated, ample data have been compiled about the circadian transcriptome, and more recently about the even larger circadian proteome [14]–[16]. These data sets, however, are directly dependent on the genome of a species and cannot be easily compared between model systems. Changes in physiology and metabolism governed by these genes and proteins, however, ultimately affect the abundance of small metabolites. Such metabolites are well conserved amongst species, which makes them promising as biomarkers in basic research and clinical investigations.

Therefore, we and others have previously characterized temporal patterns in the human metabolome using an unbiased or targeted approach that combined liquid and/or gas chromatography followed by mass spectrometry in saliva and plasma [17]–[21]. Consistently, ∼20% of the observed metabolites showed circadian variation. Sleep and food intake, however, seemed to be important co-variables [17]. Moreover, significant inter-individual variability was observed. The study of Chua and co-workers [20] found that some plasma lipids are up to 12 hours out of phase between participants.

Here, we extend the previous methodology to the breath metabolome in a proof-of-principle study with human subjects. As previously reported, we find breath to be a rich matrix for small metabolites [22], [23], some of which show a circadian rhythmic temporal profile. Thus, we provide first evidence that “breathprinting” is a feasible approach to probe circadian fluctuations in the human metabolome. Moreover, even with only a small sample size, we were able to infer time-of-day of breathing (±3 hours) from the metabolite composition of a single breath; this although inter-individual differences were sizeable. The breath metabolome is especially alluring because measurements are completely non-invasive, fast and allow for real-time analysis, with detection levels down to parts per trillion [24], [25]. Therefore, instantaneous results about circadian parameters could be provided in research or clinical applications of this technique.

## Methods

### Participants and Study Protocol

For this proof-of-principle study, breath was sampled from 3 volunteers (2 males, 1 female) of heterogeneous ethnic background. The ETH ethics commission approved the measurements (EK 2012-N-25) and all participants gave written informed consent to participate. Procedures and tests were in accordance with the Declaration of Helsinki.

Before the onset of the laboratory study, the healthy, non-smoking participants of similar age (33–38 years) were monitored by wrist actigraphy and adhered to a regular but not defined sleep schedule (S1 Fig.). None of the participants declared any drug-use and they were advised to not drink caffeinated beverages in the 3 days before or during the study. For the laboratory study, participants came into the laboratory after habitual wake-up time and at least one hour before the first sample was taken, and then stayed in the laboratory for 24 hours of continuous prolonged wakefulness. During this time, participants remained seated or walked back and forth to the breath sampling in an adjacent room. Participants were given hourly isocaloric meals (small piece of bread and ½ fruit) together with a glass of water just after breathing into the mass spectrometer. At all times, participants were indoors under constant illumination (50–200 lx, RO-1332, Rotronic, Germany) and temperature (∼21°C).

### Breath Sampling

Breath was measured in hourly intervals throughout except at 1800 due to a malfunction of the instrument. Each measurement consisted of deep exhalations. Participants were instructed to keep the pressure constant (at 10 mbar) across the sampling line during exhalation. This was achieved by feedback to the participants using a digital manometer. This process was repeated 4–7 times per participant, with the replicate measurements altogether taking typically no more than 10 minutes. S2 Fig. shows a picture of the breath analysis set-up.

### Real-time breath mass spectrometric analysis

**Figure 1:**
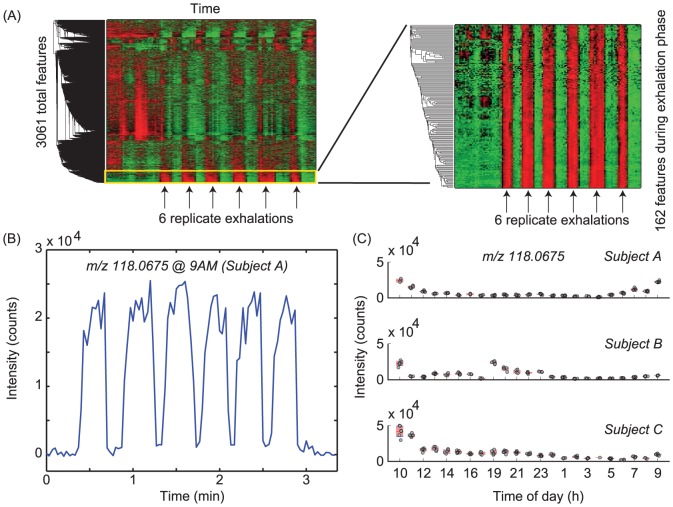
(A) Mass spectrometric data processing workflow. For every series of six exhalations (mid-exhalation indicated by arrows), the intensity of 3061 centroided mass spectra as a function of time were subjected to hierarchical cluster analysis (left). The 162 signals rising in intensity during the exhalation phase tend to group together and are further considered (zoomed view of yellow box, right-hand side). Warmer colors signify higher abundance. (B) Example of one of such compounds rising during the 6 replicate exhalations of one participant. Note that in less than 3 minutes 6 repeatable breathprints are recorded. Average signal at the plateau of the replicate exhalations were computed for all three participants, and if the intensity at all the time points was lower than 4000 counts, the feature was not considered. (C) Final data set showcasing inter- and intra- individual differences of an exhaled compound (tentatively identified as indole; *m/z* 118.0675) during 24 hours for three different participants. Note the tightly clustered replicate breath for the same clock time. Red shading signifies 95% confidence interval around the mean. The separation between hourly measurements has been arbitrarily set to ease visualization.

The experimental set-up has been presented in detail previously [22], [23], [26], [27]. Briefly, the entrance of a commercial quadrupole time-of-flight mass spectrometer (Synapt G2S, Waters, UK) was slightly modified to allow for the admission of breath samples through a heated Teflon tube. Exhaled breath was mixed with a nano-electrospray plume (water, 0.2% formic acid), whereby some compounds present in breath were readily detected in real time. The mass spectrometer was operated in the positive ion mode. The scans were recorded using the mass-lock option, using a common mass spectroscopy (MS) contaminant as lock mass (i.e., protonated phthalic anhydride, mass-to-charge-ratio (*m/z*) 149.0233 [28]). The data analysis work-flow is given in Fig. 1.

#### Raw data preprocessing

Raw mass spectrometric files collected during the 24 hours experiment were first converted into mzXML format by using the open source tool MSConverter [29], and imported into Matlab (R2013b) for further processing. Spectra were then resampled to 300,000 data points by shape-preserving piecewise cubic interpolation in the range *m/z* 52–500. All mass spectra were assembled into a single 300,000×8,798 matrix (i.e., *m/z*× scan number). Every mass spectrum was aligned using common contaminant ions appearing in the mass range of interest as reference peaks [28]. Finally, the spectra were centroided (±0.003 *m/z*), reducing as a result the matrix size to 3,061×8,798. The features above the background were filtered out by subjecting the 69 data sub-matrices (i.e., 23 hourly samples ×3 participants) to hierarchical cluster analysis. As a result, only signals that increased during the exhalations were kept for further analysis (Fig. 1A).

Then, the baseline of the time traces were corrected and signals of a minimum intensity of 4,000 counts in at least one time point were retained. A total of 111 *m/z* channels were retained after this step. The mean intensities at the plateau of each exhalation recorded during the 24 hours experiment were retrieved to construct three matrices: 132×111 (Participant A); 120×111 (Participant B); 121×111 (Participant C). Finally, the intensities of the replicates at a given time point were averaged resulting in three matrices (one for each participant) of 23×111 (hourly samples ×*m/z*). These data are available as S1 Dataset.

#### Circadian Analysis

*Principal components analysis:* The three matrices containing the signal intensities at the plateau of each of the exhalations recorded (132×111 (Participant A); 120×111 (Participant B); 121×111 (Participant C)) were autoscaled and subjected to principal components analysis (PCA) using the built-in MATLAB function.

*JTK and bootstrapping:* The proportion of circadian metabolites was estimated using a bootstrapping method as previously described [18], and cross-checked with JTK-Cycle [30]. Results of both methods were highly comparable.

*Timetable method for phase prediction:* For the time-table analysis, we used a partial least-squares (PLS) regression model to test whether breathprints could be used to predict time-of-day of breathing. We used the z-score of the average data of even times (0800, 1000, 1200,…) of all three participants as training data-set to generate a discriminant function. The first three PLS components, explaining 89% of the variance, were used to fit the training data-set. Then, this function was used to predict time-of-day of breathing from uneven times of day (0900, 1100, 1300,…) for each single participant.

## Results

We report a total of 111 features in breath with *m/z* ranging from 59 to 239. Most likely chemical formulas deduced from the recorded spectra are given in S1 Table. Amongst these, considerable intra- and inter-individual variation was found, as has been reported previously [17], [20], [23]. As an example, Fig. 1B shows the time trace of the signal at *m/z* 118.0675 (tentatively identified as indole) for one participant at 0900. Clearly, the signal increases above the background level with similar intensities in all 6 replicates. Fig. 1C shows an overview of the distinct behavior of this particular compound during the 24 hours measurement for the three participants.

Although there was satisfactory intra-individual repeatability, levels between individuals were at different absolute levels. For example, the first measurement collected at around 1000, showed a mean intensity of 2.4×10^4^, 2.2×10^4^ and 4.2×10^4^ counts for participants A, B and C, respectively. While the trends were different over the day for the three of them (most apparent for Participant B), all were at minimal levels around 0300–0400. At 0400, the signal intensity of indole at 0400 was 792, 1.5×10^3^ and 4.84×10^3^ for the three participants, implying a signal drop of an up to 30-fold. S3 Fig. shows additional examples of selected ions that exhibited distinct inter-subject rhythm.

**Figure 2:**
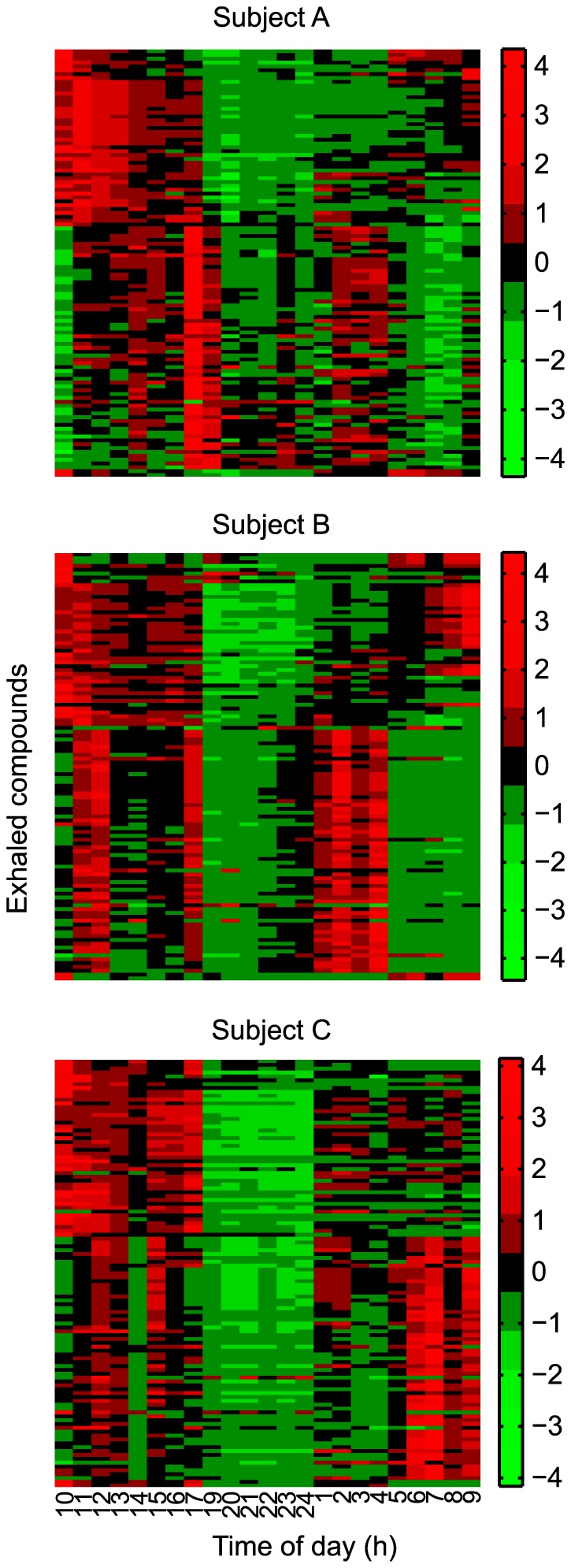
Individual heat-maps for all three participants at all times of all 111 features that were extracted from the spectrograms. Order of metabolites in all three heat maps based on results of clustering of metabolites in Participant A.

To further investigate which features of the 111 breath signals showed a circadian pattern, we applied the JTK algorithm [30] (S2 Table). As result, we obtained that 40, 54 and 40 compounds (participants A, B and C, respectively, i.e., 36–49%) showed a significant (p <0.01) circadian modulation. The number of common features identified as rhythmic between individuals was moderate for participants A and B (28 in total), whereas Participant C showed a lower overlap (2 features with A and 16 with B). There was also some inter-individual difference in phasing as can be assessed from the heat maps of all metabolites ordered by phase in Participant A (Fig. 2).

**Figure 3:**
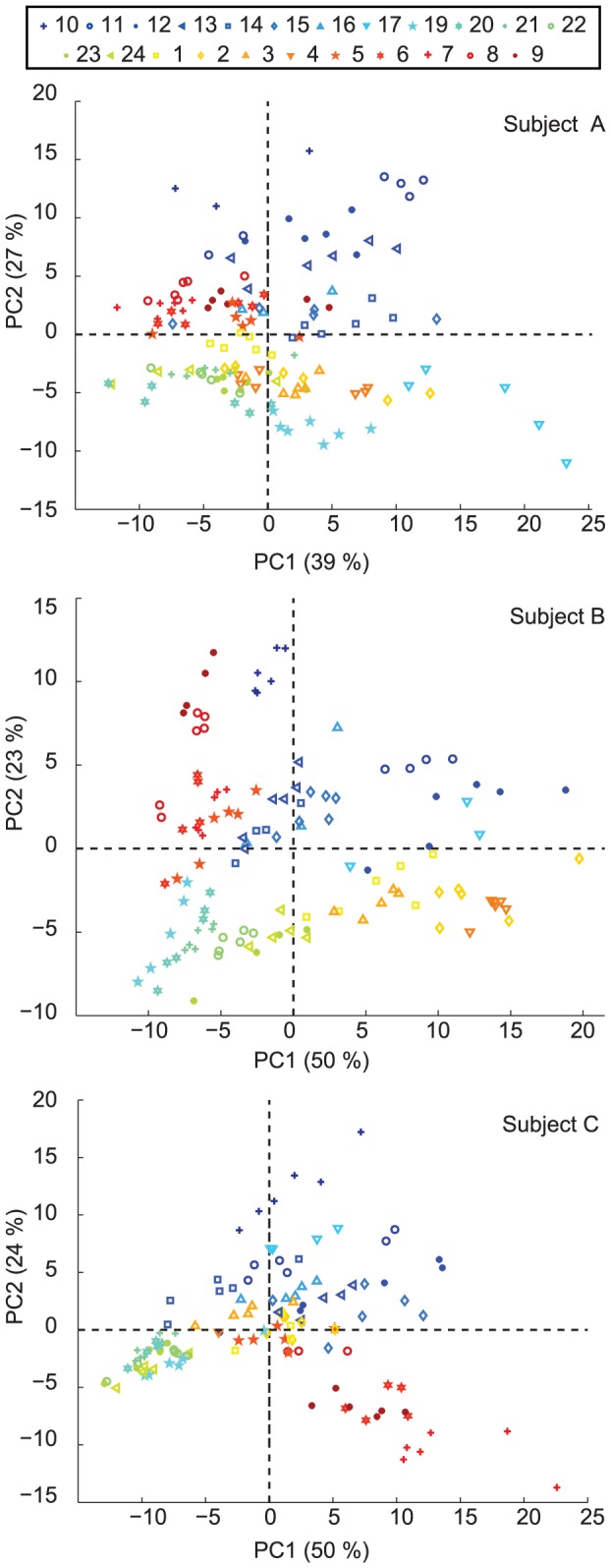
PCA score plot of the breath mass spectral fingerprints. Replicate measurements tend to cluster together, while different time points tend to occupy different areas on the plot.

In an effort to enable an independent global visualization of the temporal evolution of the breath profiles we reduced the dimensionality of the breathprints by subjecting the mass spectra to PCA. Fig. 3 shows the corresponding score plot for the first two principal components; explaining 66, 73 and 74% of the total variation for participants A, B and C respectively. Replicate breaths of one time of day tend to cluster together. Likewise, similar times of day are more closely related than ones further apart. Overall, the segregation pattern suggests that the breath at a particular time of day has a unique composition. This was apparent for all the three participants, despite the clear rhythmic differences suggested by the individual time traces of each of the compounds shown in Fig. 2, and is also very much in line with previous results spanning only from morning to late afternoon [27].

**Figure 4:**
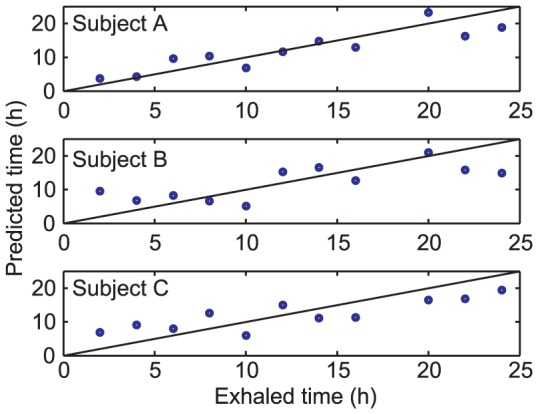
Time-table method for time-stamping the breathprint. Correlation of time of actual breathing (exhaled time) to prediction of PLS for each participant. Average error was 2.7±1.8 h, 4±2.6 h and 4±1 h for participants A, B and C, respectively. Correlation coefficients between predicted and exhaled time were 0.8971, 0.7442 and 0.8499 for participants A, B and C, respectively.

To further capitalize on the good temporal separation of breathprinting, we conducted partial least-squares (PLS) regression to establish a time-table method for the breath metabolome. Data of all 111 features of all three participants were averaged for each feature used as a training set. Then, time-of-day was predicted from the PLS function. The time of day of breathing was predicted within 2.7±1.8 h, 4.0±2.6 h and 4.0±1.0 h (mean ± SD) of the actual time based on the training set of “population averages” for participants A, B and C, respectively. The corresponding correlation coefficients between predicted and exhaled time were also reasonable: 0.8971, 0.7442 and 0.8499 for participants A, B and C, respectively. Data for all three participants are shown in Fig. 4.

In an effort to verify the striking dip in various features in the late day, we replicated the around the clock measurement on a different QTOF mass spectrometer (Triple TOF, AB Sciex, Canada, S1 Experiment), and found even more discernible features (around 400; S4 Fig.). While comparing data between different mass spectrometers may be difficult, we are certain to have identified examples in the data of the two mass spectrometers and they do indeed have a comparable time course (S5 Fig.).

## Discussion

We report on the first attempt to capture circadian modulations in human metabolites by real-time breath analysis. Our data suggest that non-invasive real-time mass spectrometric "breathprinting" has significant potential to track time of day changes. The results are in line with previous reports [27] and extend them from differences between morning and afternoon to a full circadian cycle. They suggest that breathprinting has potential to become a useful tool in tracking and understanding the influence of circadian clock on metabolism. Indeed, alternative real-time gas analysis techniques like selected ion flow tube-mass spectrometry (SIFT-MS) and proton transfer reaction-mass spectrometry (PTR-MS) have previously shown promise as a valuable approach to track physiological changes during extended periods of time [31] and diet challenges [32], [33] and even during complete sleeping phases [34] using breath. Similarly, pioneering real-time breath analysis studies using atmospheric pressure chemical ionization mass spectrometry (APCI-MS) [35], [36], found quite individual-specific daily-patterns of ammonia in breath [37]. The present study reinforces the notion that, in combination with suitable analytical tools, the mass spectrometric real-time analysis of exhaled metabolites may successfully contribute to gain insights into biological processes in fields such as chronobiology. In this regard, it is important to stress that compound identification is a crucial step to achieve this goal. For unambiguous structural elucidation, traditional 2-dimensional separation of breath compounds (e.g., gas chromatography-mass spectrometry) remains the most powerful approach. Using this technique hundreds of compounds have been identified in breath [38], [39] some of which may be of relevance in chronobiology. Our results clearly suggests that the effort to identify the detected breath-metabolites is worthwhile undertaking; Identification of these compounds will be a key step to relate our results to other well-known plasma and saliva circadian and sleep related metabolites [40]. Apart from previously identified acetone (m/z 59.05) and indole (m/z 118.07), the fingerprinting strategy used here to describe overall 24-hours changes does not allow for the unambiguous identification of compounds, but it does provide their most likely molecular formulas (S1 Table) [41]. Of note, however, the technique used in this study (secondary electrospray ionization (SESI) [42]–[55]), coupled with high resolution commercial mass spectrometer allows for the detection of targeted metabolites with high confidence and in real-time. For example, the potential to measure fatty acids in breath in negative-ion mode [56] seems particularly promising as this group of molecules has been previously shown to be of interest for metabolic phenotyping [20]. Thus, in future studies, more features will be measurable by measuring in positive as well as negative ion mode within a few minutes. To further validate this method, it will be critical to establish the relationship of the breathprint to other current circadian phase-markers like melatonin or cortisol as well as other potential or already confirmed biomarkers for metabolism and/or disease. Once these control experiments have been conducted, however, the presented method together with further technical advances may significantly expand the possibilities to further explore the human metabolome in basic research and clinical applications.

Due to the practical advantages of analyzing breath (i.e., non-invasiveness and online real-time analysis) compared to sampling of saliva or blood, breathprinting could be a major step toward using metabolomics in real-world experimental settings and even clinical practice. Thus, in concert with other recent advances in the understanding of the modulation of metabolism through the clock using metabolomic and lipodomic approaches in blood [17]–[21], our method has the potential to become state-of-the-art. However, further constant routine and “real world” experiments are needed to better define proportion and magnitude of intra- and inter-individual variation; especially parameters like the timing of the rhythms or circadian phase, sleep pressure and food intake seem of special practical and clinical importance in these experiments. For example, it has recently been suggested that sleep deprivation or mistimed sleep has a major impact on the metabolome [21] as well as the transcriptome [57], [58]. These further experiments should also help to explain the higher proportion of cycling features that we observed in breath (ca. 40%) compared to previous saliva and blood sampling studies that reported roughly 20% [17]–[19]. We are, however, fully in line with the recent results of Davies and colleagues that found 45% of blood metabolites to be rhythmic in total sleep deprivation [21] using targeted analysis. This might suggest that not the matrix that is analyzed but the method of analysis could be important for the proportion of observed rhythmic metabolites.

One of the interesting problems of chronobiology that is highly relevant for the translation of its accumulating results into the clinic is the accurate determination of internal circadian phase (or body time). For example, clinical studies could time the administration of drugs to a certain phase in the circadian cycle, because pharmacokinetics and dynamics are known to vary with circadian phase resulting in greater efficacy at particular circadian phases [59]. Variations in internal phase between individuals can be quite large and depend on the timing of sleep and wake, which is in turn driven by whether an individual is a morning or evening type (i.e., chronotype). To wit, individuals with extreme chronotypes can exhibit internal circadian phase differences of as much as 12 hours at the same time of day [20], [60], [61], making the determination of internal circadian phase non-trivial. Indeed, there have been previous attempts to predict internal phase from gene expression in mouse liver, and; a single sample measurement was able to predict internal time with 30 min accuracy [62]. Later, this method was adapted to predict phase from mouse serum metabolites [63] and this has been extended to human blood metabolites [19]. However, in the latter case, the precision went down to more than 3 hours. Of course, our proof-of-principle study cannot address this issue comprehensively due to its small sample size and the heterogeneous “study-population” and the less than ideal technical limitations of the protocol posed by the circumstances with regards to illumination levels and co-sampling of known phase markers. Even with such limitations, our data, analyzed by PCA and PLS, can roughly determine time-of-day (clock time) from the composition of breath. This suggests that breathprinting might allow assessment of circadian phase with one single sample with medically useful precision. Moreover it would allow non-invasive – and even more interestingly – instantaneous phase estimation, which would be highly advantageous in experimental or clinical settings. So far, prediction of body time with a single sample measure using a time-table method has been shown to be imprecise in human subjects. This is probably mostly due to inter-individual differences absolute levels of metabolites. Interestingly, these absolute levels of blood metabolite have recently been linked to genetic variances and loci associated with diseases in genome wide association studies [64], [65]. Of note, however, none of these studies, each of which identified a number of genome-metabolome associations, has taken circadian variation in levels of metabolites into account. Given the sizable time-of-day dependent variation that we and others have observed in some metabolites, further studies should carefully check possible time-of-day variation when searching for biomarkers of disease or significant genomic associations with the metabolome.

Correct and immediate identification of overall circadian phase of an individual would have multiple advantages, e.g., for the correct timing of drug delivery or determination of rhythmic hormone levels. Also, chronotypes could be easily and objectively determined. This is of interest because certain chronotypes have been linked to lower resilience against shift-work or higher incidence of depression [66], [67]. Moreover, information about the degree of overall rhythmicity or disruption of rhythmicity alone would have diagnostic value since disruption of clock circadian timing has been linked to metabolic and psychiatric diseases. For example, acute and chronic “social” jet-lag, defined as significant variation between sleep timing on weekends and weekdays, has been associated with metabolic syndrome and diabetes [68]. Moreover, circadian disruption of behavior is observed in some psychiatric diseases like schizophrenia [3]. Here, a simple one-time-point measure of internal synchrony could prove a valuable diagnostic marker.

In conclusion, utilizing novel real-time breathprinting we showed that the breath metabolome has a rich composition of small molecules, and about 40% of the 111 detected features showed significant circadian modulation. Therefore we conclude that this method seems ideally suited to investigate the effect of time-of-day on the human metabolome.

## Supporting Information

S1 Fig **Normalized double-plotted actograms of wrist actigraphy (Actiwatch worn on the wrist of the non-dominant hand) from participants A (left), B (middle) and C (right).** Red underlined area signifies the 24 hrs of the breath sampling on day 7 and 8 of the recording. Grey shaded area signifies malfunction of the actigraph.
(PDF)

S2 Fig **Commercial atmospheric pressure ionization mass spectrometer modified to analyze exhaled breath in real-time (symbolic picture only, not a participant of the study).**
(PDF)

S3 Fig **Breath composition varies substantially depending on the time-of-day.** The figure shows the intensity of six exemplary breath signals (*m/z* listed on top of the figure) over the course of the day for three participants (top A, middle B, bottom C). Each point represents the raw measurements at a given time. Red shading signifies 95% confidence interval around the mean. Note the inter-individual differences of the participants A, B and C.
(PDF)

S4 Fig **Individual heat-maps for Participant B and an additional volunteer (D) as measured in a different instrument (Sciex's TripleTOF).** The relative intensity of ∼400 features during 15 hours is plot.
(PDF)

S5 Fig **Temporal trends of exhaled metabolites are reasonably reproducible.** The figure compares the relative breath intensity of eight compounds exhaled by participant B in two different mass spectrometers (Water's synapt in blue; Sciex's Tripletof in red).
(PDF)

S1 Table **Most likely chemical formulas of observed features.**
(PDF)

S2 Table **Results of JTK-cycle analysis for all participants and all features.**
(PDF)

S1 Experiment **Experimental material and methods for replication experiment.**
(PDF)

S1 Dataset **The intensities of all replicates at a given time point in three matrices (one for each participant) of 23×111 (hourly samples ×** ***m/z*** **).**
(XLSX)
