# Supplementary material for: Circadian Variation of the Human Metabolome Captured by Real-Time Breath Analysis
Source: PLoS One. 2014 Dec 29;9(12):e114422. doi: 10.1371/journal.pone.0114422 (PMC4278702; doi:10.1371/journal.pone.0114422)
Supplement: S1 Fig — Normalized double-plotted actograms of wrist actigraphy (Actiwatch worn on the wrist of the non-dominant hand) from participants A (left), B (middle) and C (right). Red underlined area signifies the 24 hrs of the breath sampling on day 7 and 8 of the recording. Grey shaded area signifies malfunction of the actigraph. (PDF) [file pone.0114422.s001.pdf]

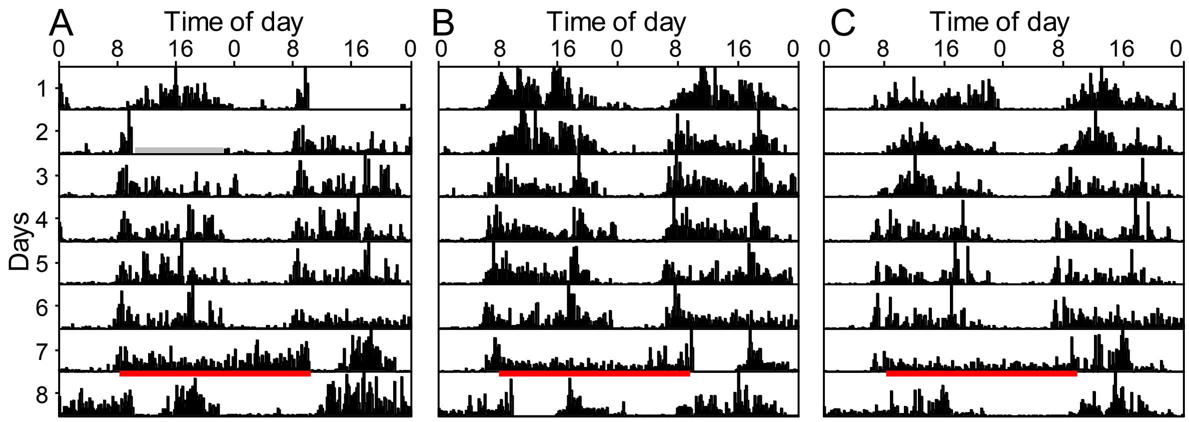

Figure S1. Normalized double-plotted actograms of wrist actigraphy (Actiwatch worn on the wrist of the non-dominant hand) from participants A (left), B (middle) and C (right). Red underlined area signifies the 24 hrs of the breath sampling on day 7 and 8 of the recording. Grey shaded area signifies malfunction of the actigraph.
