# Supplementary material for: Circadian Variation of the Human Metabolome Captured by Real-Time Breath Analysis
Source: PLoS One. 2014 Dec 29;9(12):e114422. doi: 10.1371/journal.pone.0114422 (PMC4278702; doi:10.1371/journal.pone.0114422)
Supplement: S2 Fig — Commercial atmospheric pressure ionization mass spectrometer modified to analyze exhaled breath in real-time (symbolic picture only, not a participant of the study). (PDF) [file pone.0114422.s002.pdf]

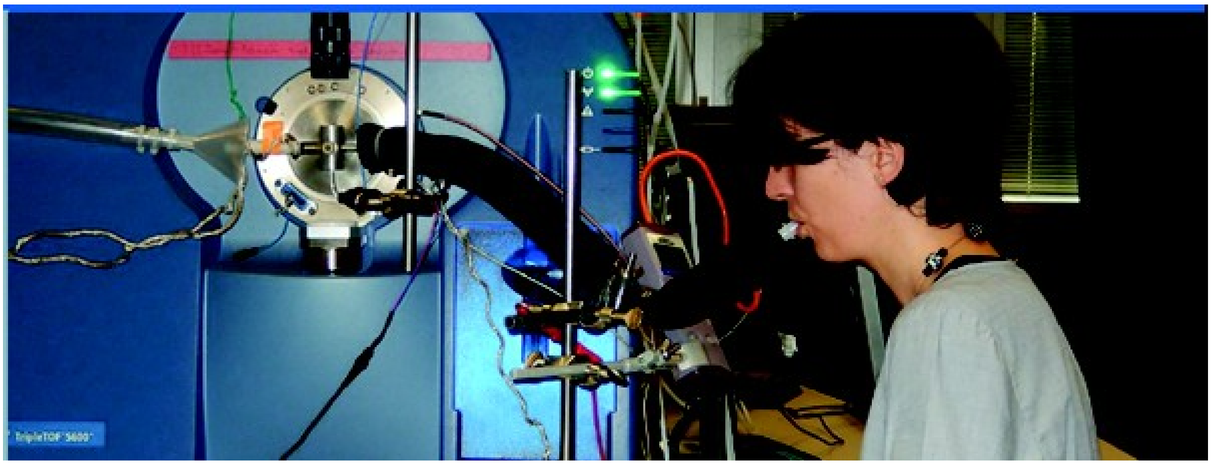

Figure S2. Commercial atmospheric pressure ionization mass spectrometer modified to analyze exhaled breath in real-time (symbolic picture only, not a participant of the study).
