# Supplementary material for: Circadian Variation of the Human Metabolome Captured by Real-Time Breath Analysis
Source: PLoS One. 2014 Dec 29;9(12):e114422. doi: 10.1371/journal.pone.0114422 (PMC4278702; doi:10.1371/journal.pone.0114422)
Supplement: S3 Fig — Breath composition varies substantially depending on the time-of-day. The figure shows the intensity of six exemplary breath signals (m/z listed on top of the figure) over the course of the day for three participants (top A, middle B, bottom C). Each point represents the raw measurements at a given time. Red shading signifies 95% confidence interval around the mean. Note the inter-individual differences of the participants A, B and C. (PDF) [file pone.0114422.s003.pdf]

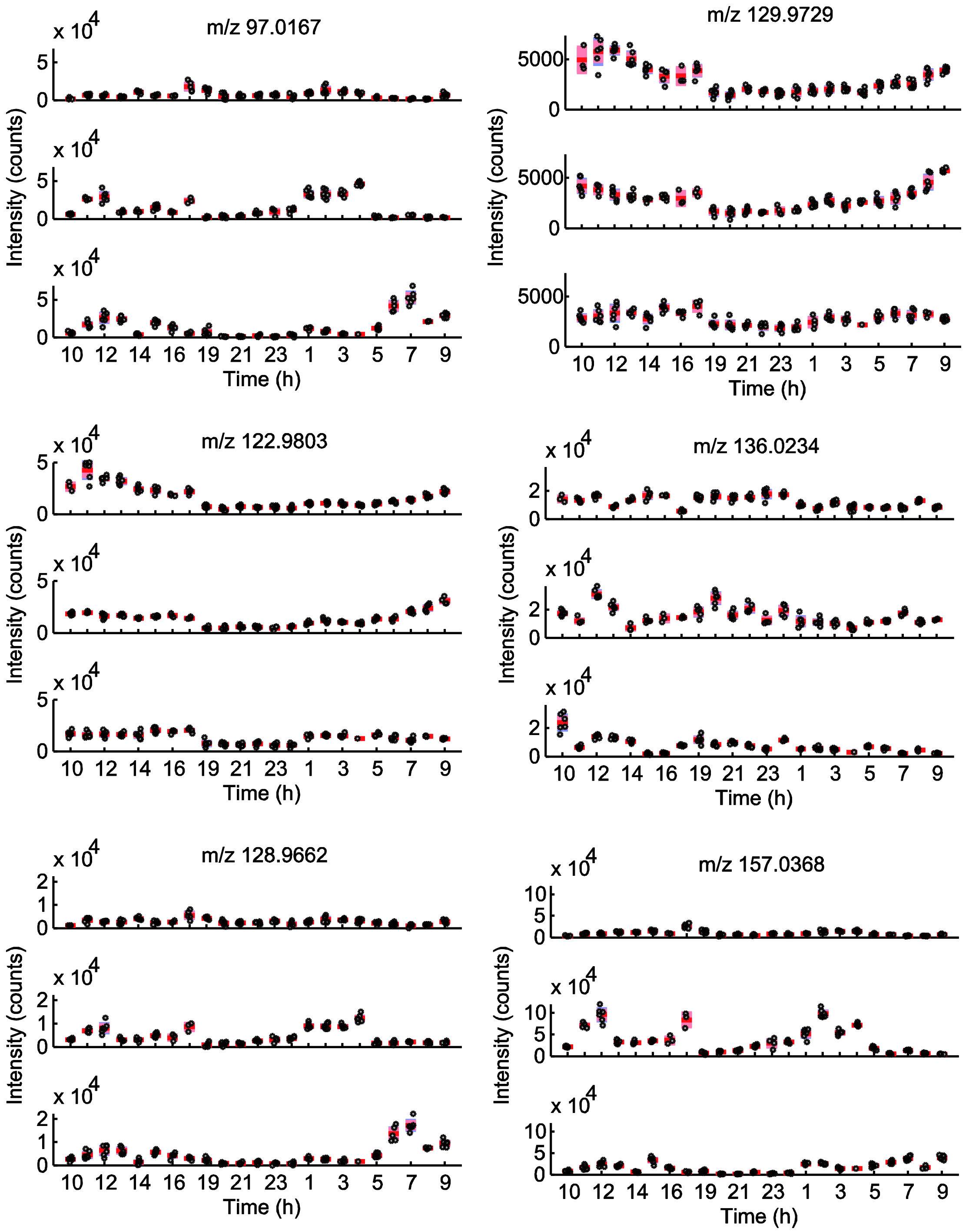

Figure S3. Breath composition varies substantially depending on the time-of-day. The figure shows the intensity of six exemplary breath signals (*m/z* listed on top of the figure) over the course of the day for three participants (top A, middle B, bottom C). Each point represents the raw measurements at a given time. Red shading signifies 95% confidence interval around the mean. Note the inter-individual differences of the participants A, B and C.
