# Supplementary material for: Circadian Variation of the Human Metabolome Captured by Real-Time Breath Analysis
Source: PLoS One. 2014 Dec 29;9(12):e114422. doi: 10.1371/journal.pone.0114422 (PMC4278702; doi:10.1371/journal.pone.0114422)
Supplement: S4 Fig — Individual heat-maps for Participant B and an additional volunteer (D) as measured in a different instrument (Sciex's TripleTOF). The relative intensity of ∼400 features during 15 hours is plot. (PDF) [file pone.0114422.s004.pdf]

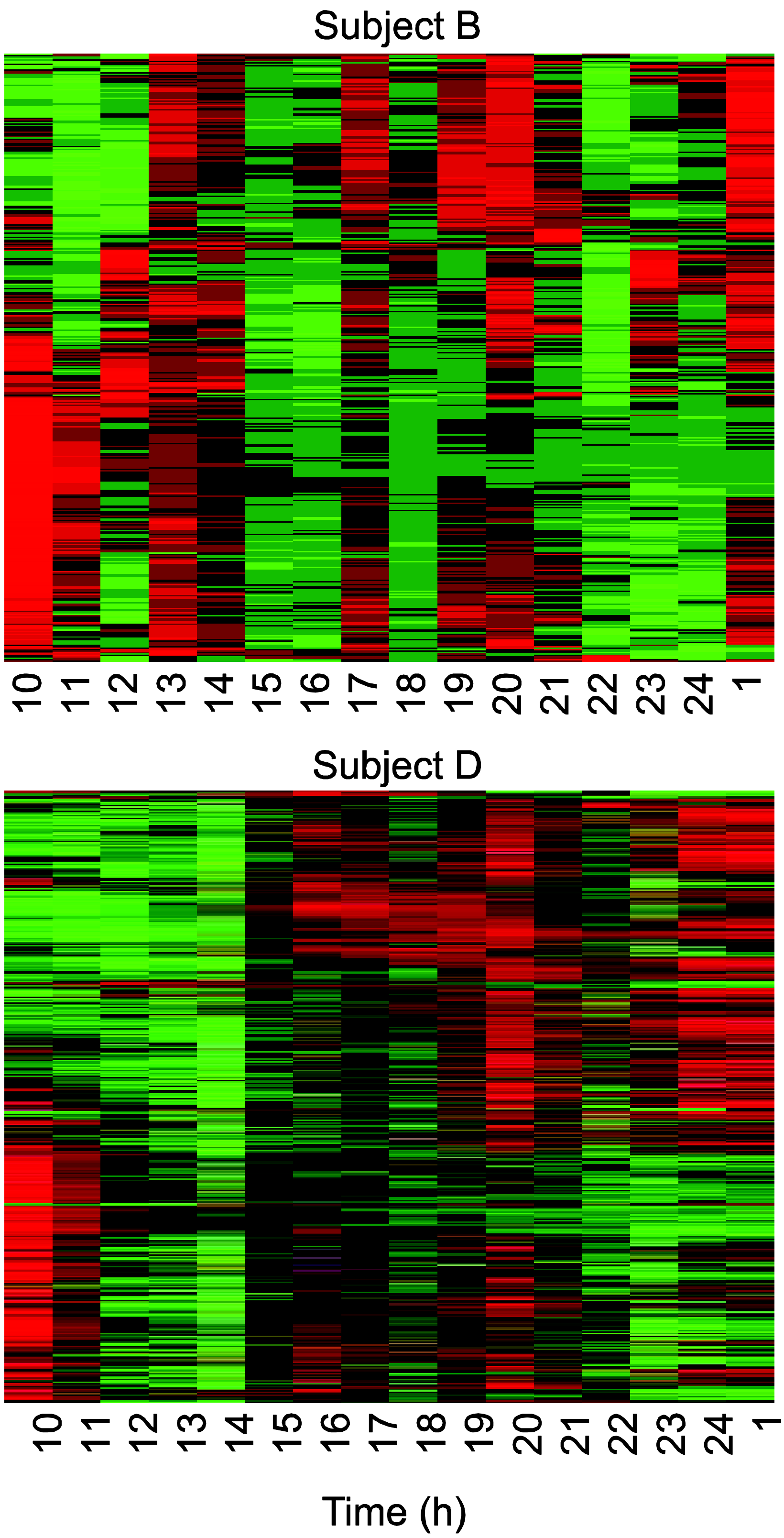

Figure S4. Individual heat-maps for **Participant** B and an additional volunteer (D) as measured in a different instrument (Sciex´s TripleTOF). The relative intensity of ~ 400 features during 15 hours is plot.
