# Supplementary material for: Circadian Variation of the Human Metabolome Captured by Real-Time Breath Analysis
Source: PLoS One. 2014 Dec 29;9(12):e114422. doi: 10.1371/journal.pone.0114422 (PMC4278702; doi:10.1371/journal.pone.0114422)
Supplement: S5 Fig — Temporal trends of exhaled metabolites are reasonably reproducible. The figure compares the relative breath intensity of eight compounds exhaled by participant B in two different mass spectrometers (Water's synapt in blue; Sciex's Tripletof in red). (PDF) [file pone.0114422.s005.pdf]

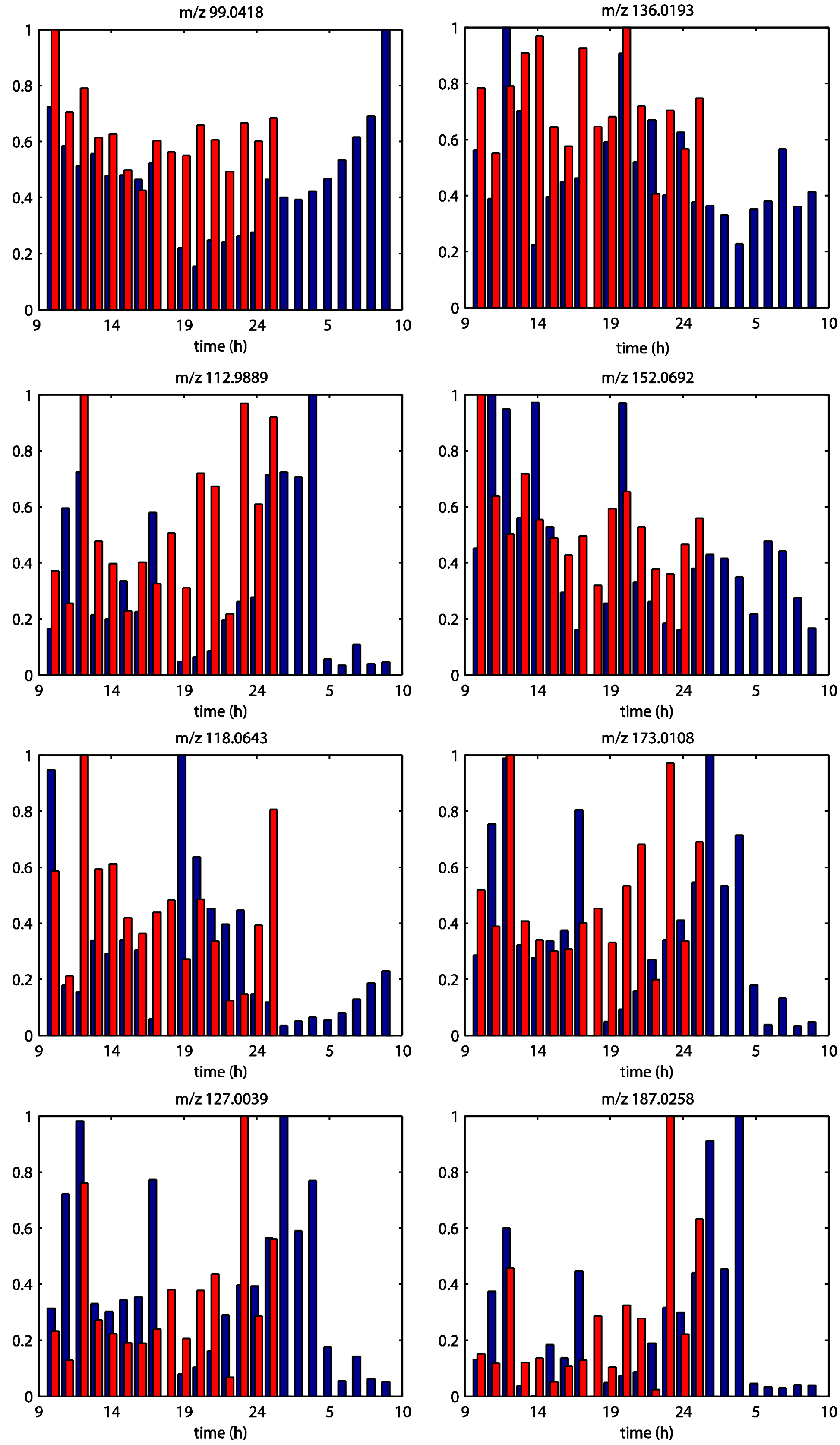

Figure S5. Temporal trends of exhaled metabolites are reasonably reproducible. The figure compares the relative breath intensity of eight compounds exhaled by participant B in two different mass spectrometers (Water's synapt in blue; Sciex's Tripletof in red).
