# Supplementary material for: Circadian Variation of the Human Metabolome Captured by Real-Time Breath Analysis
Source: PLoS One. 2014 Dec 29;9(12):e114422. doi: 10.1371/journal.pone.0114422 (PMC4278702; doi:10.1371/journal.pone.0114422)
Supplement: S1 Table — Most likely chemical formulas of observed features. (PDF) [file pone.0114422.s006.pdf]

Table S1. Most likely chemical formulas of observed features.

Input mass	Adduct	Mass	Dppm	Formula
59.05583	[M+H]+	58.041865	113	C3H6O
99.03254	[M+H-H2O]+	116.0334254	18	C2H4N4O2
109.0671	[M+H]+	108.0575118	21	C7H8O
109.0671	[M+H-H2O]+	126.0680796	16	C7H10O2
111.0324	[M+H-H2O]+	128.0334254	15	C3H4N4O2
112.034	[M+H]+	111.032029	47	C5H5NO2
112.9939	[M+H]+	111.9830293	31	CH4O4S
113.0293	[M+H]+	112.027278	46	C4H4N2O2
113.0293	[M+H-H2O]+	130.0266087	48	C5H6O4
113.0475	[M+H]+	112.0346709	49	C6H8S
113.0475	[M+H]+	112.038511	15	C3H4N4O
113.0475	[M+H-H2O]+	130.0452356	44	C6H10OS
113.0475	[M+H-H2O]+	130.0530982	25	C8H6N2
115.0266	[M+H]+	114.0139355	46	C5H6OS
115.0266	[M+H-H2O]+	132.0245002	42	C5H8O2S
117.0589	[M+H]+	116.047345	36	C5H8O3
117.0589	[M+H-H2O]+	134.0579088	31	C5H10O4
117.0589	[M+H-H2O]+	134.059246	20	C6H6N4
118.0675	[M+H]+	117.057849	20	C8H7N
119.0308	[M+H]+	118.0200835	28	C3H6N2OS
119.0308	[M+H]+	118.02661	25	C4H6O4
119.0308	[M+H-H2O]+	136.0346709	9	C8H8S
119.0308	[M+H-H2O]+	136.037175	30	C4H8O5
119.0308	[M+H-H2O]+	136.0380418	37	C5H12S2
119.0308	[M+H-H2O]+	136.038511	41	C5H4N4O
122.9803	[M+H-H2O]+	139.9779439	41	C2H4O5S
122.9803	[M+H-H2O]+	139.9788123	34	C3H8S3
127.009	[M+H]+	125.998682	23	C2H6O4S
127.009	[M+H-H2O]+	144.0067416	39	C6H8S2
127.026	[M+H]+	126.0139355	37	C6H6OS
129.0419	[M+H]+	128.0334254	9	C3H4N4O2
129.0419	[M+H]+	128.040819	48	C5H8N2S
129.0419	[M+H-H2O]+	146.0401503	34	C6H10O2S
129.0419	[M+H-H2O]+	146.0480128	26	C8H6N2O
131.0321	[M+H]+	130.020085	36	C4H6N2OS
131.0321	[M+H]+	130.0266087	13	C5H6O4
131.0321	[M+H-H2O]+	148.0371734	17	C5H8O5
131.0321	[M+H-H2O]+	148.0380418	24	C6H12S2
136.0234	[M+H]+	135.0142699	13	C7H5NS
136.0234	[M+NH4]+	117.9902232	4	C3H2O5
141.024	[M+H]+	140.0109586	40	C6H4O4
141.024	[M+H]+	140.022192	38	C5H4N2O3
141.024	[M+H-H2O]+	158.0215233	36	C6H6O5
141.024	[M+H-H2O]+	158.032758	42	C5H6N2O4
141.042	[M+H]+	140.0295856	36	C7H8OS
141.042	[M+H]+	140.040819	43	C6H8N2S
141.042	[M+NH4]+	123.0142699	43	C6H5NS
141.042	[M+H-H2O]+	158.0401503	32	C7H10O2S
141.042	[M+H-H2O]+	158.0439901	5	C4H6N4O3
149.0979	[M+H]+	148.088815	12	C10H12O

Input mass	Adduct	Mass	Dppm	Formula
149.0979	[M+H]+	148.0921858	10	C7H16OS
149.0979	[M+NH4]+	131.058226	39	C5H9NO3
149.0979	[M+NH4]+	131.0694765	36	C4H9N3O2
149.0979	[M+H-H2O]+	166.0966944	26	C6H10N6
149.0979	[M+H-H2O]+	166.099371	8	C10H14O2
150.9758	[M+H-H2O]+	167.982378	25	C3H5O6P
151.0254	[M+H]+	150.0139355	27	C8H6OS
151.0254	[M+H-H2O]+	168.0245005	24	C8H8O2S
151.0254	[M+H-H2O]+	168.028341	1	C5H4N4O3
152.0636	[M+H]+	151.04941	45	C5H5N5O
152.0636	[M+H]+	151.063284	45	C8H9NO2
152.0636	[M+NH4]+	134.032758	19	C3H6N2O4
152.0636	[M+NH4]+	134.0367794	46	C8H6O2
152.0636	[M+H-H2O]+	169.073894	49	C8H11NO3
152.9904	[M+H]+	151.9779439	33	C3H4O5S
152.9904	[M+H]+	151.9874598	28	C3H5O5P
152.9904	[M+H-H2O]+	169.9885086	30	C3H6O6S
152.9904	[M+H-H2O]+	169.9980245	31	C3H7O6P
154.9869	[M+H]+	153.9758354	24	C3H6O3S2
155.0573	[M+H]+	154.0452356	30	C8H10OS
155.0573	[M+NH4]+	137.0299199	41	C7H7NS
155.0573	[M+H-H2O]+	172.0524268	49	C11H8O2
155.0573	[M+H-H2O]+	172.0636629	23	C10H8N2O
157.0368	[M+H]+	156.0245002	31	C7H8O2S
157.0368	[M+H]+	156.034193	29	C8H9ClO
157.0368	[M+H]+	156.0357336	39	C6H8N2OS
157.0368	[M+H-H2O]+	174.0316941	49	C10H6O3
157.0368	[M+H-H2O]+	174.0429274	21	C9H6N2O2
158.1563	[M+NH4]+	140.1201151	14	C9H16O
159.0327	[M+H]+	158.0215233	24	C6H6O5
159.0327	[M+H]+	158.0327567	46	C5H6N2O4
159.0327	[M+NH4]+	141.0062076	46	C5H3NO4
159.0327	[M+H-H2O]+	176.032088	21	C6H8O6
159.0327	[M+H-H2O]+	176.040819	33	C9H8N2S
159.0327	[M+H-H2O]+	176.0433214	49	C5H8N2O5
166.9713	[M+H-H2O]+	183.977293	19	C3H5O7P
168.9858	[M+H]+	167.9728586	33	C3H4O6S
168.9858	[M+H]+	167.9823744	22	C3H5O6P
168.9858	[M+H-H2O]+	185.992943	26	C3H7O7P
168.9858	[M+H-H2O]+	185.9944531	34	C5H3ClN4O2
170.9648	[M+H-H2O]+	187.9635562	23	C3H8O3S3
171.052	[M+H]+	170.0401503	26	C8H10O2S
171.052	[M+H]+	170.0439901	4	C5H6N4O3
171.052	[M+H]+	170.051385	38	C7H10N2OS
171.052	[M+NH4]+	153.0190943	5	C3H8NO4P
171.052	[M+H-H2O]+	188.0473441	43	C11H8O3
171.052	[M+H-H2O]+	188.0585775	22	C10H8N2O2
172.99	[M+H-H2O]+	189.993594	4	C6H6O5S
173.0139	[M+H]+	172.0007878	33	C6H4O6
173.0139	[M+H]+	172.0136745	40	C3H9O6P
173.0139	[M+H-H2O]+	190.011355	30	C6H6O7
180.9854	[M+H-H2O]+	197.9834232	26	C4H6O7S

Input mass	Adduct	Mass	Dppm	Formula
180.9854	[M+H-H2O]+	197.9929391	26	C4H7O7P
185.068	[M+H]+	184.0524295	44	C12H8O2
185.068	[M+H]+	184.0558003	26	C9H12O2S
185.068	[M+H]+	184.0636629	15	C11H8N2O
185.068	[M+NH4]+	167.0252285	48	C4H9NO4S
185.068	[M+NH4]+	167.026567	41	C5H5N5S
185.068	[M+NH4]+	167.0347443	3	C4H10NO4P
185.068	[M+NH4]+	167.036464	12	C3H9N3O3S
185.068	[M+NH4]+	167.0404846	34	C8H9NOS
185.068	[M+H-H2O]+	202.0629942	41	C12H10O3
185.068	[M+H-H2O]+	202.0718581	6	C4H15N2O5P
185.068	[M+H-H2O]+	202.0742276	18	C11H10N2O2
185.068	[M+H-H2O]+	202.0782503	40	C16H10
187.009	[M+H-H2O]+	204.0189365	38	C8H9ClO4
187.0284	[M+H]+	186.0164379	25	C7H6O6
187.0284	[M+H-H2O]+	204.0270026	22	C7H8O7
187.0284	[M+H-H2O]+	204.0357336	24	C10H8N2OS
189.0601	[M+H]+	188.0473441	28	C11H8O3
189.0601	[M+H]+	188.0585775	30	C10H8N2O2
189.0601	[M+NH4]+	171.0217694	23	C5H11Cl2NO
189.0601	[M+NH4]+	171.0353992	48	C7H9NO2S
189.0601	[M+H-H2O]+	206.0579088	26	C11H10O4
189.0601	[M+H-H2O]+	206.069143	33	C10H10N2O3
194.9997	[M+H-H2O]+	212.0004107	10	C6H4N4O3S
196.9806	[M+H-H2O]+	213.9870688	18	C7H6N2O2S2
196.9806	[M+H-H2O]+	213.987858	22	C4H7O8P
197.0401	[M+H]+	196.0232546	48	C6H4N4O4
197.0401	[M+H]+	196.0371734	22	C9H8O5
197.0401	[M+H-H2O]+	214.0412129	8	C8H10N2O3S
197.0401	[M+H-H2O]+	214.0477381	24	C9H10O6
197.0401	[M+H-H2O]+	214.0490755	31	C10H6N4O2
197.0401	[M+H-H2O]+	214.052444	48	C7H10N4O2S
197.0671	[M+H]+	196.0524295	37	C13H8O2
197.0671	[M+H]+	196.059641	0	C7H8N4O3
197.0671	[M+H]+	196.067035	36	C9H12N2OS
197.0671	[M+NH4]+	179.0252285	40	C5H9NO4S
197.0671	[M+NH4]+	179.0404846	36	C9H9NOS
197.0671	[M+NH4]+	179.042987	49	C5H9NO6
197.0671	[M+H-H2O]+	214.0629942	34	C13H10O3
197.0671	[M+H-H2O]+	214.066365	17	C10H14O3S
197.0671	[M+H-H2O]+	214.0702048	1	C7H10N4O4
197.0671	[M+H-H2O]+	214.0776	39	C9H14N2O2S
197.9826	[M+H-H2O]+	214.9807768	23	C8H6ClNO2S
198.038	[M+H]+	197.0324223	8	C8H7NO5
198.038	[M+H]+	197.0357932	25	C5H11NO5S
198.038	[M+H-H2O]+	215.033091	38	C10H5N3O3
203.0035	[M+H]+	201.993594	12	C7H6O5S
203.0236	[M+H]+	202.0113525	24	C7H6O7
203.0236	[M+NH4]+	184.9994077	47	C3H7NO6S
203.0236	[M+H-H2O]+	220.0219172	21	C7H8O8
205.0289	[M+H]+	204.027005	26	C7H8O7
205.0289	[M+H-H2O]+	222.0350649	16	C11H10O3S

Input mass	Adduct	Mass	Dppm	Formula
206.9989	[M+H-H2O]+	224.0078043	29	C9H8N2OS2
213.0452	[M+H]+	212.032088	27	C9H8O6
213.0452	[M+H]+	212.0473441	44	C13H8O3
213.0452	[M+NH4]+	195.020146	41	C5H9NO5S
213.0452	[M+H-H2O]+	230.057891	46	C13H10O4
214.9911	[M+H]+	213.9870688	15	C7H6N2O2S2
214.9911	[M+H-H2O]+	232.0041587	47	C8H8O6S
219.0858	[M+H]+	218.0691422	42	C11H10N2O3
219.0858	[M+H]+	218.072513	27	C8H14N2O3S
219.0858	[M+H]+	218.072515	27	C8H14N2O3S1
219.0858	[M+H]+	218.073161	24	C16H10O
219.0858	[M+H]+	218.0790382	2	C9H14O6
219.0858	[M+H-H2O]+	236.079708	40	C11H12N2O4
219.0858	[M+H-H2O]+	236.0830777	24	C8H16N2O4S
219.0858	[M+H-H2O]+	236.0837296	21	C16H12O2
219.0858	[M+H-H2O]+	236.090941	10	C10H12N4O3
219.0858	[M+H-H2O]+	236.094963	29	C15H12N2O
225.0123	[M+H]+	224.0078043	12	C9H8N2OS2
239.024	[M+H-H2O]+	256.0371734	43	C14H8O5
