# Supplementary material for: Circadian Variation of the Human Metabolome Captured by Real-Time Breath Analysis
Source: PLoS One. 2014 Dec 29;9(12):e114422. doi: 10.1371/journal.pone.0114422 (PMC4278702; doi:10.1371/journal.pone.0114422)
Supplement: S2 Table — Results of JTK-cycle analysis for all participants and all features. (PDF) [file pone.0114422.s007.pdf]

**Table S2.** Results of JTK-cycle analysis for all participants and all features.

| | *p-value* | | | Period (PER) | | | Phase (LAG) | | | Amplitude (AMP) | | |
|---|---|---|---|---|---|---|---|---|---|---|---|---|
| m/z | A | B | C | A | B | C | A | B | C | A | B | C |
| 129.951 | *3.62E-05* | 5.35E-01 | 1.00E+00 | 19 | 16 | 16 | 0 | 6 | 6 | 789 | 532 | 170 |
| 127.952 | *1.21E-04* | 1.00E+00 | 1.00E+00 | 19 | 16 | 16 | 0 | 6 | 6 | 920 | 358 | 176 |
| 125.955 | *1.05E-03* | 3.57E-01 | 1.00E+00 | 19 | 11 | 16 | 0 | 10 | 6 | 1171 | 922 | 355 |
| 68.987 | *5.13E-03* | *5.31E-04* | 7.49E-02 | 23 | 22 | 17 | 1 | 15 | 2 | 2057 | 2071 | 3663 |
| 149.098 | *9.20E-03* | *5.13E-03* | 2.11E-02 | 23 | 21 | 23 | 1 | 2 | 2 | 447 | 383 | 186 |
| 118.068 | 3.56E-02 | 5.87E-02 | *9.20E-03* | 23 | 19 | 23 | 1 | 8 | 5 | 2471 | 4993 | 5922 |
| 139.971 | 3.57E-01 | 1.00E+00 | *2.35E-06* | 18 | 16 | 23 | 1 | 6 | 1 | 321 | 162 | 1144 |
| 117.059 | *2.78E-03* | 1.22E-02 | 1.22E-02 | 23 | 23 | 23 | 1 | 6 | 6 | 581 | 520 | 548 |
| 166.971 | *7.51E-04* | *2.37E-05* | 1.50E-01 | 23 | 22 | 23 | 1 | 1 | 1 | 7440 | 6334 | 7511 |
| 168.966 | *3.84E-06* | *6.16E-06* | 5.87E-02 | 23 | 21 | 23 | 2 | 1 | 1 | 4309 | 2563 | 2274 |
| 122.980 | *9.77E-06* | *2.37E-05* | 4.38E-01 | 23 | 21 | 23 | 2 | 2 | 2 | 8274 | 5969 | 2919 |
| 127.026 | *9.77E-06* | *2.86E-07* | 1.20E-01 | 23 | 22 | 23 | 2 | 1 | 2 | 3916 | 3371 | 2552 |
| 124.978 | *2.37E-05* | *5.47E-05* | 2.90E-01 | 23 | 21 | 13 | 2 | 1 | 5 | 5640 | 2949 | 3662 |
| 126.976 | *2.37E-05* | *1.53E-05* | 1.00E+00 | 23 | 20 | 16 | 2 | 2 | 6 | 3530 | 2492 | 795 |
| 142.970 | *2.37E-05* | *8.19E-05* | 1.61E-02 | 23 | 21 | 22 | 2 | 2 | 2 | 2157 | 2356 | 2392 |
| 119.031 | *2.37E-05* | *3.62E-05* | 6.52E-01 | 23 | 23 | 13 | 2 | 12 | 5 | 1519 | 1363 | 1511 |
| 59.056 | *1.78E-04* | *2.03E-03* | 5.87E-02 | 23 | 20 | 12 | 2 | 2 | 5 | 840 | 438 | 687 |
| 83.038 | *3.62E-05* | *1.42E-06* | 1.00E+00 | 23 | 23 | 16 | 2 | 22 | 7 | 3955 | 2097 | 713 |
| 170.965 | *3.62E-05* | *2.86E-07* | 3.57E-01 | 23 | 21 | 23 | 2 | 2 | 1 | 2132 | 1633 | 1277 |
| 125.979 | *8.19E-05* | *8.19E-05* | 1.00E+00 | 23 | 22 | 16 | 2 | 1 | 6 | 1002 | 899 | 297 |
| 128.027 | *8.19E-05* | *1.42E-06* | 7.90E-01 | 23 | 22 | 23 | 2 | 1 | 2 | 709 | 599 | 549 |
| 138.976 | *1.21E-04* | *1.47E-03* | 4.58E-02 | 23 | 22 | 22 | 2 | 1 | 2 | 5214 | 4655 | 5468 |
| 120.966 | *1.21E-04* | *3.62E-05* | 6.52E-01 | 23 | 22 | 23 | 2 | 1 | 1 | 2006 | 1634 | 1580 |
| 99.033 | *1.21E-04* | *2.35E-06* | 1.00E+00 | 23 | 23 | 16 | 2 | 12 | 6 | 1289 | 680 | 345 |
| 122.962 | *5.31E-04* | *8.19E-05* | 1.50E-01 | 23 | 21 | 23 | 2 | 2 | 1 | 1172 | 1143 | 675 |

| | *p-value* | | | Period (PER) | | | Phase (LAG) | | | Amplitude (AMP) | | |
|---|---|---|---|---|---|---|---|---|---|---|---|---|
| m/z | A | B | C | A | B | C | A | B | C | A | B | C |
| 219.086 | *1.47E-03* | 1.00E+00 | *3.79E-03* | 23 | 16 | 23 | 2 | 6 | 1 | 218 | 126 | 332 |
| 214.034 | *1.47E-03* | 1.00E+00 | *2.03E-03* | 23 | 16 | 23 | 2 | 6 | 3 | 139 | 145 | 339 |
| 72.942 | 2.75E-02 | 5.87E-02 | *2.78E-03* | 23 | 16 | 18 | 2 | 2 | 2 | 2742 | 2910 | 4075 |
| 158.156 | 3.56E-02 | 9.49E-02 | 1.88E-01 | 18 | 18 | 23 | 2 | 1 | 2 | 978 | 1389 | 581 |
| 114.988 | *1.78E-04* | 1.20E-01 | 1.00E+00 | 22 | 23 | 16 | 2 | 21 | 8 | 2632 | 2863 | 1705 |
| 112.971 | *6.16E-06* | *1.05E-03* | 1.00E+00 | 22 | 23 | 16 | 2 | 12 | 6 | 2379 | 753 | 365 |
| 129.973 | *6.16E-06* | *6.16E-06* | 1.61E-02 | 22 | 23 | 18 | 2 | 12 | 3 | 1114 | 817 | 821 |
| 84.931 | *6.89E-03* | 1.61E-02 | 6.52E-01 | 14 | 12 | 15 | 2 | 3 | 2 | 2939 | 3052 | 6433 |
| 89.943 | 9.49E-02 | 3.57E-01 | 2.90E-01 | 16 | 15 | 23 | 2 | 2 | 2 | 3866 | 7643 | 11201 |
| 205.029 | 1.50E-01 | 7.49E-02 | 2.11E-02 | 23 | 23 | 23 | 2 | 2 | 2 | 411 | 717 | 1726 |
| 99.938 | 1.88E-01 | 1.50E-01 | 1.00E+00 | 14 | 23 | 16 | 2 | 1 | 6 | 485 | 638 | 513 |
| 90.951 | 4.38E-01 | 2.90E-01 | 2.90E-01 | 16 | 12 | 23 | 2 | 3 | 2 | 1211 | 591 | 1979 |
| 198.038 | 6.52E-01 | 1.00E+00 | 9.49E-02 | 23 | 16 | 23 | 2 | 6 | 1 | 283 | 256 | 436 |
| 107.953 | 9.53E-01 | 7.90E-01 | 3.57E-01 | 15 | 12 | 23 | 2 | 3 | 2 | 950 | 600 | 1497 |
| 130.982 | *3.62E-05* | 5.87E-02 | 1.20E-01 | 22 | 23 | 13 | 2 | 21 | 6 | 2083 | 1434 | 1516 |
| 197.040 | 1.61E-02 | 1.88E-01 | *6.89E-03* | 23 | 23 | 23 | 3 | 2 | 3 | 386 | 389 | 716 |
| 141.024 | 7.49E-02 | 1.00E+00 | *2.58E-04* | 23 | 16 | 23 | 3 | 6 | 1 | 439 | 522 | 881 |
| 86.997 | 1.20E-01 | *1.47E-03* | 1.20E-01 | 13 | 13 | 23 | 3 | 3 | 2 | 1376 | 2756 | 2658 |
| 100.941 | 2.34E-01 | 2.11E-02 | 9.53E-01 | 13 | 13 | 23 | 3 | 3 | 2 | 3955 | 3265 | 7971 |
| 98.945 | 6.52E-01 | 2.11E-02 | 1.00E+00 | 13 | 12 | 16 | 3 | 3 | 5 | 3912 | 5294 | 8464 |
| 109.067 | 1.88E-01 | 1.00E+00 | 7.90E-01 | 23 | 16 | 8 | 4 | 10 | 1 | 444 | 121 | 450 |
| 111.032 | 2.75E-02 | *6.89E-03* | 1.50E-01 | 11 | 13 | 18 | 5 | 3 | 2 | 2639 | 5281 | 4361 |
| 112.034 | 3.56E-02 | *1.47E-03* | 2.75E-02 | 11 | 13 | 18 | 5 | 2 | 2 | 286 | 1056 | 825 |
| 113.029 | 7.49E-02 | *7.51E-04* | *9.20E-03* | 11 | 13 | 18 | 5 | 2 | 3 | 314 | 950 | 766 |
| 113.048 | 1.20E-01 | *5.31E-04* | 7.49E-02 | 11 | 13 | 18 | 5 | 2 | 2 | 274 | 443 | 559 |
| 156.985 | 1.50E-01 | 1.22E-02 | 1.22E-02 | 11 | 13 | 21 | 5 | 3 | 2 | 1613 | 5221 | 4231 |
| 195.000 | 1.00E+00 | *6.89E-03* | *1.05E-03* | 16 | 13 | 18 | 5 | 2 | 3 | 172 | 810 | 707 |

| | *p-value* | | | Period (PER) | | | Phase (LAG) | | | Amplitude (AMP) | | |
|---|---|---|---|---|---|---|---|---|---|---|---|---|
| m/z | A | B | C | A | B | C | A | B | C | A | B | C |
| 239.024 | 1.00E+00 | *9.20E-03* | 4.38E-01 | 16 | 14 | 18 | 5 | 2 | 3 | 52 | 335 | 265 |
| 197.067 | 1.00E+00 | 2.90E-01 | 2.11E-02 | 16 | 13 | 14 | 5 | 4 | 5 | 766 | 4281 | 2969 |
| 157.037 | *8.19E-05* | *5.13E-03* | 4.58E-02 | 11 | 13 | 23 | 5 | 3 | 22 | 4197 | 8331 | 9445 |
| 154.987 | *1.21E-04* | *5.13E-03* | 3.56E-02 | 11 | 13 | 21 | 5 | 3 | 12 | 1863 | 5158 | 3265 |
| 152.990 | *1.05E-03* | *1.47E-03* | 5.87E-02 | 11 | 13 | 18 | 5 | 3 | 3 | 3290 | 7765 | 5088 |
| 188.987 | *1.47E-03* | *2.03E-03* | 1.61E-02 | 11 | 13 | 23 | 5 | 3 | 22 | 882 | 1510 | 2150 |
| 198.977 | *2.03E-03* | *6.89E-03* | 1.50E-01 | 11 | 13 | 23 | 5 | 3 | 22 | 1467 | 3784 | 3482 |
| 196.981 | *3.79E-03* | 1.22E-02 | 1.88E-01 | 11 | 13 | 23 | 5 | 3 | 22 | 2418 | 6449 | 5959 |
| 142.981 | *3.79E-03* | *6.89E-03* | 3.56E-02 | 11 | 13 | 23 | 5 | 3 | 22 | 871 | 2003 | 1964 |
| 127.009 | 5.87E-02 | *3.79E-03* | 1.22E-02 | 11 | 13 | 18 | 5 | 2 | 2 | 3084 | 5927 | 7046 |
| 173.014 | 5.87E-02 | *1.47E-03* | 7.49E-02 | 11 | 13 | 23 | 5 | 2 | 22 | 662 | 1506 | 3279 |
| 159.033 | 1.50E-01 | *3.79E-03* | *5.13E-03* | 11 | 13 | 23 | 5 | 2 | 22 | 418 | 1140 | 1143 |
| 150.976 | 1.88E-01 | *9.20E-03* | 4.58E-02 | 11 | 13 | 21 | 5 | 2 | 12 | 496 | 2002 | 916 |
| 98.018 | 4.38E-01 | *1.47E-03* | *3.72E-04* | 11 | 12 | 18 | 5 | 4 | 2 | 125 | 1080 | 885 |
| 168.986 | 1.00E+00 | *2.78E-03* | *2.78E-03* | 16 | 13 | 20 | 5 | 2 | 2 | 211 | 1918 | 1201 |
| 180.985 | 1.00E+00 | 1.61E-02 | *2.58E-04* | 16 | 14 | 19 | 5 | 2 | 3 | 240 | 933 | 1329 |
| 199.981 | 2.11E-02 | 1.61E-02 | 1.20E-01 | 11 | 13 | 23 | 6 | 2 | 22 | 332 | 833 | 500 |
| 197.983 | 4.58E-02 | *5.13E-03* | 3.56E-02 | 11 | 13 | 19 | 6 | 2 | 2 | 194 | 565 | 539 |
| 153.986 | 2.90E-01 | 1.50E-01 | *3.72E-04* | 10 | 15 | 20 | 6 | 2 | 2 | 167 | 858 | 1427 |
| 185.068 | 4.38E-01 | 2.75E-02 | *6.89E-03* | 10 | 13 | 17 | 6 | 2 | 4 | 105 | 1496 | 751 |
| 97.017 | 5.35E-01 | *6.89E-03* | *1.47E-03* | 10 | 13 | 18 | 6 | 3 | 3 | 213 | 5299 | 4257 |
| 171.052 | 6.52E-01 | 1.61E-02 | *9.20E-03* | 10 | 13 | 23 | 6 | 3 | 22 | 218 | 174 | 1458 |
| 213.045 | 1.00E+00 | 9.53E-01 | 1.61E-02 | 16 | 12 | 15 | 6 | 5 | 5 | 294 | 2889 | 2332 |
| 225.012 | 3.56E-02 | *2.78E-03* | *9.20E-03* | 10 | 13 | 16 | 6 | 2 | 5 | 181 | 587 | 380 |
| 170.981 | 1.00E+00 | *5.13E-03* | *7.51E-04* | 16 | 13 | 19 | 6 | 2 | 3 | 58 | 803 | 873 |
| 146.977 | 1.00E+00 | 2.34E-01 | *2.03E-03* | 16 | 13 | 17 | 6 | 2 | 4 | 274 | 1391 | 992 |
| 206.999 | 1.00E+00 | 2.75E-02 | *1.78E-04* | 16 | 13 | 18 | 6 | 2 | 3 | 122 | 505 | 463 |

| | *p-value* | | | Period (PER) | | | Phase (LAG) | | | Amplitude (AMP) | | |
|---|---|---|---|---|---|---|---|---|---|---|---|---|
| m/z | A | B | C | A | B | C | A | B | C | A | B | C |
| 131.032 | *5.13E-03* | *2.78E-03* | 2.34E-01 | 10 | 13 | 23 | 7 | 2 | 22 | 502 | 1162 | 829 |
| 141.042 | 3.57E-01 | 3.56E-02 | *5.47E-05* | 23 | 14 | 17 | 7 | 2 | 4 | 730 | 919 | 1179 |
| 155.057 | 9.53E-01 | 3.56E-02 | *1.05E-03* | 23 | 13 | 17 | 7 | 2 | 4 | 646 | 856 | 1358 |
| 152.064 | 1.00E+00 | 1.00E+00 | 1.00E+00 | 16 | 16 | 16 | 7 | 6 | 8 | 103 | 637 | 244 |
| 155.987 | 5.87E-02 | *6.89E-03* | *2.03E-03* | 9 | 13 | 18 | 7 | 2 | 2 | 39 | 808 | 965 |
| 83.002 | 5.35E-01 | *1.47E-03* | *1.78E-04* | 9 | 13 | 23 | 7 | 3 | 22 | 51 | 1805 | 4209 |
| 214.991 | 7.90E-01 | 1.22E-02 | *2.58E-04* | 9 | 13 | 18 | 7 | 2 | 3 | 344 | 524 | 457 |
| 200.975 | 9.53E-01 | 1.61E-02 | 3.56E-02 | 23 | 13 | 19 | 7 | 2 | 2 | 1951 | 4990 | 4429 |
| 172.990 | 1.00E+00 | 3.56E-02 | *2.03E-03* | 16 | 13 | 17 | 7 | 2 | 4 | 0 | 615 | 733 |
| 129.042 | 7.49E-02 | *2.03E-03* | *1.47E-03* | 23 | 13 | 18 | 8 | 2 | 2 | 484 | 1759 | 1524 |
| 189.060 | 7.90E-01 | *5.13E-03* | *2.58E-04* | 9 | 13 | 17 | 8 | 2 | 4 | 83 | 554 | 455 |
| 128.966 | 1.00E+00 | 3.56E-02 | *1.05E-03* | 16 | 13 | 17 | 8 | 3 | 4 | 33 | 1686 | 2380 |
| 136.023 | 1.00E+00 | 1.00E+00 | 1.00E+00 | 16 | 16 | 16 | 8 | 7 | 7 | 1173 | 1994 | 1047 |
| 172.981 | 7.49E-02 | 1.22E-02 | *1.21E-04* | 23 | 13 | 18 | 8 | 2 | 3 | 196 | 774 | 393 |
| 187.009 | 1.00E+00 | 3.56E-02 | 7.49E-02 | 16 | 14 | 18 | 8 | 2 | 3 | 18 | 480 | 120 |
| 122.944 | 1.00E+00 | *6.89E-03* | *1.78E-04* | 16 | 13 | 18 | 8 | 2 | 3 | 152 | 1014 | 1157 |
| 151.025 | 1.00E+00 | 1.00E+00 | 1.00E+00 | 16 | 16 | 16 | 8 | 5 | 5 | 455 | 733 | 559 |
| 203.004 | 1.00E+00 | 1.50E-01 | 7.49E-02 | 16 | 13 | 23 | 8 | 2 | 21 | 45 | 657 | 289 |
| 160.993 | 1.50E-01 | 1.22E-02 | *2.78E-03* | 23 | 13 | 18 | 9 | 2 | 3 | 88 | 1187 | 579 |
| 112.994 | 1.00E+00 | *6.89E-03* | *2.78E-03* | 16 | 13 | 18 | 9 | 3 | 3 | 633 | 5556 | 3800 |
| 124.941 | 1.00E+00 | *3.79E-03* | *8.19E-05* | 16 | 13 | 19 | 9 | 2 | 3 | 34 | 794 | 706 |
| 114.952 | 1.00E+00 | 6.52E-01 | 3.56E-02 | 16 | 13 | 17 | 9 | 2 | 4 | 323 | 926 | 698 |
| 82.985 | 1.00E+00 | *1.05E-03* | 1.88E-01 | 16 | 14 | 23 | 9 | 2 | 21 | 27 | 533 | 1024 |
| 115.027 | 2.75E-02 | 2.75E-02 | *5.13E-03* | 21 | 13 | 18 | 10 | 2 | 3 | 936 | 2666 | 1360 |
| 203.024 | 3.56E-02 | *5.13E-03* | *3.62E-05* | 21 | 13 | 17 | 10 | 2 | 4 | 267 | 346 | 519 |
| 183.991 | 1.00E+00 | 9.53E-01 | 1.50E-01 | 16 | 23 | 13 | 10 | 17 | 5 | 184 | 1013 | 578 |
| 187.028 | 4.38E-01 | 1.00E+00 | *2.58E-04* | 21 | 16 | 21 | 10 | 9 | 12 | 191 | 547 | 214 |

| | *p-value* | | | Period (PER) | | | Phase (LAG) | | | Amplitude (AMP) | | |
|---|---|---|---|---|---|---|---|---|---|---|---|---|
| m/z | A | B | C | A | B | C | A | B | C | A | B | C |
| 98.978 | 1.50E-01 | 1.20E-01 | *2.03E-03* | 20 | 15 | 23 | 11 | 8 | 22 | 908 | 2326 | 2067 |
| 101.011 | 2.34E-01 | 5.87E-02 | *2.03E-03* | 18 | 13 | 23 | 11 | 2 | 23 | 329 | 785 | 1360 |
| 144.999 | 6.52E-01 | 2.75E-02 | 9.49E-02 | 20 | 13 | 23 | 12 | 2 | 20 | 553 | 640 | 827 |
| 85.963 | *9.77E-06* | 6.52E-01 | 1.00E+00 | 20 | 23 | 16 | 20 | 0 | 6 | 1393 | 611 | 462 |
| 83.964 | *1.53E-05* | 1.00E+00 | 1.00E+00 | 20 | 16 | 16 | 20 | 6 | 6 | 1647 | 124 | 710 |
