# Supplementary material for: Circadian Variation of the Human Metabolome Captured by Real-Time Breath Analysis
Source: PLoS One. 2014 Dec 29;9(12):e114422. doi: 10.1371/journal.pone.0114422 (PMC4278702; doi:10.1371/journal.pone.0114422)
Supplement: S1 Experiment — Experimental material and methods for replication experiment. (PDF) [file pone.0114422.s008.pdf]

Methods S1.

We confirmed the daily pattern of changes in breath metabolites in an independent experiment using a different mass spectrometer (AB Sciex Triple TOF). In this case, Participant B and one additional volunteer (Participant D) conducted a similar protocol during 15 hours (1000-0100) of extended wakefulness in order to cover the most interesting phases as suggested by the data obtained previously. The results yielded a higher number of features in breath, which probably was due to the higher resolution of this instrument. In total, ~400 features could be found in participants B and D. No circadian analysis was conducted due to the limited time-window.

Instead, the time series of Participants B was subjected to hierarchical cluster analysis. Figure S4 shows the resulting heat-map and the corresponding one for Participant D, suggesting again significant time-dependent changes in breath's chemical composition. Furthermore, the same pattern of change could be observed in multiple features as compared to the previous measurements from Participant B (Figure S5). Thus, suggesting robust and stable daily variation in these metabolites.
